# High Prevalence of Multidrug-Resistant Bacteria in the Trachea of Intensive Care Units Admitted Patients: Evidence from a Bangladeshi Hospital

**DOI:** 10.3390/antibiotics13010062

**Published:** 2024-01-08

**Authors:** Sabrina Haque, Akash Ahmed, Nazrul Islam, Fahim Kabir Monjurul Haque

**Affiliations:** 1 Microbiology Program, Department of Mathematics and Natural Sciences, BRAC University, Dhaka 1212, Bangladesh; haque.sabrina123@gmail.com (S.H.); akash.ahmed@bracu.ac.bd (A.A.); 2 IBN SINA Diagnostic and Imaging Center, Dhaka 1209, Bangladesh; nazrul111015@gmail.com

**Keywords:** multidrug-resistant bacteria, ICU patient, effective antibiotic

## Abstract

Recent research has shown that antibiotic-resistant microorganisms are becoming more prevalent in intensive care units (ICUs) at an exponential rate. Patients in the ICU can get infected by pathogens due to invasive operation procedures and critical health conditions. This study primarily emphasized tracheal samples from ICU patients due to their reliance on ventilators, increasing their susceptibility to Ventilator-Associated Pneumonia (VAP). Moreover, the rise of multidrug-resistant (MDR) pathogens makes treatment strategies more challenging for these patients. In this study, we tested 200 tracheal specimens to determine the prevalence of microorganisms and analyzed the antibiotic susceptibility of these isolates against regular antibiotics, including 4th generation drugs. Among the 273 isolates, 81% were gram-negative bacteria, 10% were gram-positive bacteria, and 9% were fungi. The most prevalent gram-negative bacteria were *Acinetobacter* spp. (34%), *Klebsiella* spp. (22%), *Pseudomonas* spp. (14%), and *Escherichia coli* (9.2%). The most prevalent gram-positive bacteria were *Staphylococcus aureus* (5.9%), and the fungi were *Candida* spp. (7.3%). Among the most prevalent bacteria, except *Staphylococcus aureus* isolates, around 90% were resistant to multiple drugs, whereas 60% of *Acinetobacter* spp. and *Pseudomonas* spp. were extensively drug resistant. Sensitivity analysis against the gram-negative and gram-positive drug panel using a one-way ANOVA test followed by Tukey’s post hoc test showed that in the in vitro assay, colistin was the most effective antibiotic against all gram-negative bacteria. In contrast, linezolid, vancomycin, and fusidic acid were most effective against all gram-positive bacteria. Regular monitoring of nosocomial infections and safe management of highly resistant bacteria can help prevent future pandemics.

## 1. Introduction

Antibiotic resistance is becoming a serious problem worldwide, including in the Asia–Pacific, Latin American, Middle Eastern, European, and North American regions [1]. However, Southeast Asia and the Middle East are two regions that contribute a high burden of antimicrobial resistance because people can easily buy antibiotics without any prescription [1,2]. However, additional factors could contribute to these regions’ heightened prevalence of antimicrobial resistance. Bonten and Mascini identified four primary driving factors contributing to the emergence and subsequent dissemination of multidrug-resistant (MDR) microorganisms: (1) the creation of resistant strains; (2) the favoring of resistant strains through selection; (3) the introduction of resistant strains; and (4) the wide distribution of resistant strains. These changeable and interrelated factors should be particularly emphasized as motivations for addressing the proliferation of antimicrobial resistance [3]. Variations in the prevalence of antimicrobial resistance (AMR) among countries are influenced by various factors. These factors encompass the extent of antibiotic usage, access to clean water and proper sanitation, vaccination coverage rates, the presence of quality healthcare services, and accessibility to high-grade medical products [4].

Considering it an immense threat to public health, the WHO has also categorized the spread of antibiotic resistance as one of the three most severe threats in the twenty-first century [5].

Though antibiotic-resistant microorganisms are ubiquitous, recent research showed an exponential increase in intensive care units (ICU) [6]. ICUs are one of the significant facilitators in creating, disseminating, and magnifying drug-resistant organisms at healthcare facilities [6]. Patients who are critically ill and admitted to the ICU are at a greater risk of acquiring infections from resistant strains [7]. The rate of nosocomial infections in the ICU is about 2–5 times higher than in the general hospital population [4]. Among the frequently occurring diseases in the ICU, lower respiratory tract infections are the most common. Around 10–25% of ICU patients acquired these infections, resulting in a colossal mortality toll ranging from 22 to 71% [7]. The most common cause of developing such infections is multidrug-resistant microorganisms. Recent studies have reported that most of the frequently isolated microorganisms from ICUs are multidrug resistant, and these are common phenomena all around the globe [7,8,9,10,11,12,13]. A study of Nepalese ICUs reported that 83.1% of the isolated organisms from patient tracheal aspirates were multidrug resistant [8].

It has been observed that ICUs are one of the significant sources of both gram-positive and gram-negative bacteria that cause infectious diseases [14]. Since the last decade, *Pseudomonas* spp., *Acinetobacter* spp., *Klebsiella* spp., *Citrobacter* spp., *Escherichia coli*, and *Candida* spp. have been found consistently at ICUs [7,8,10,11,15,16,17,18]. Three categories of gram-negative bacteria have been addressed as high-priority pathogens because of their ubiquitous presence in healthcare facilities: (i) extended-spectrum beta-lactamase (ESBL)-producing *Escherichia coli* and *Klebsiella* spp., (ii) multidrug-resistant (MDR) *Pseudomonas* spp., and (iii) carbapenem-resistant *Acinetobacter* spp. [19].

Antibiotic resistance varies over time, geographic area, availability, local production, and uses of antimicrobials. It can even vary in different healthcare settings within a particular area [7,20]. Therefore, it is essential to regularly monitor healthcare settings, especially ICUs, to prevent or reduce the spread of multidrug-resistant opportunistic pathogens. Thus, this study aimed to identify the most prevalent microorganisms in the trachea and their susceptibility to commonly used antimicrobials in a local hospital’s ICU in Dhaka City, Bangladesh.

## 2. Results

Tracheal aspiration was collected from the ET tube of 200 patients. Among them, 123 (61.5%) were male, and 77 (38.5%) were female, aged between 13 and 99 years. Their age-wise distribution is shown in Table 1 The majority of the participants in this study were old adults aged 60 or more (64%) (Table 1).

**Figure 1:**
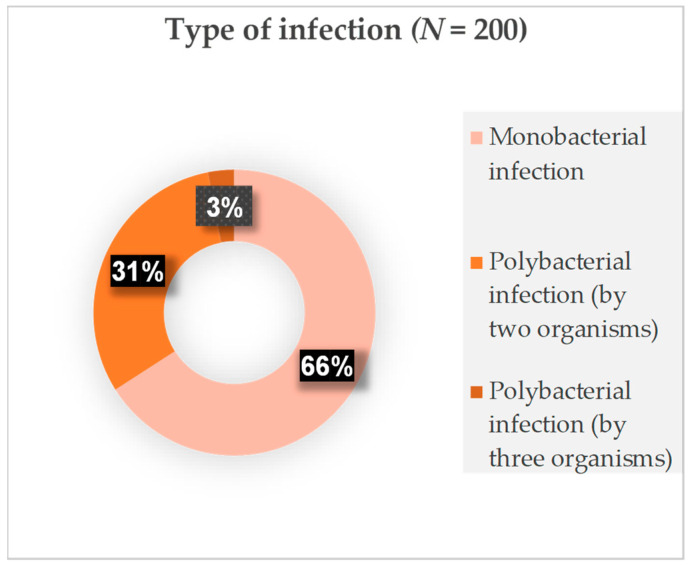
Distribution of microorganisms. The figure specifies in percentages whether the infection was monobacterial or polybacterial. It also includes the cases with fungal infection.

A total of 273 isolates were isolated from the trachea of the ICU patients. Of the 200 patients, 132 (66%) had a monobacterial infection. The remaining 68 (34%) patients exhibited a polybacterial infection (Figure 1).

Of all the bacteria isolated, gram-negative bacteria were the majority at 81%, whereas gram-positive organisms accounted for a mere 10%. *Acinetobacter* spp. and *Klebsiella* spp. were found to be the most common gram-negative bacteria, constituting more than 56% of all microorganisms. On the other hand, *Enterobacter* spp., *Moraxella* spp., *Proteus* spp., Saprophytic fungi, and *Staphylococcus* spp. were the least prevalent, accounting for a total of 3.65% of all organisms. Compared to other microorganisms, the prevalence of *Candida* spp. was low, accounting for around 7% of the isolated organisms (Table 2). Moreover, this study found that about 61% of *Acinetobacter* spp. and 70% of *Klebsiella* spp. infections occurred among individuals aged 60 years or older. This study also found that more than 50% of *E. coli*, *Pseudomonas* spp., and *Staphylococcus aureus* infections were prevalent in the age group of 60 years and above 60 (Table 1).

Based on frequency, isolated organisms were categorized into two groups: most prevalent organisms (Table 3) and least prevalent organisms (Table S1). The top five most prevalent microorganisms included *E. coli*, *Acinetobacter* spp., *Klebsiella* spp., and *Pseudomonas* spp., which are gram-negative, and *Staphylococcus aureus*, which is gram-positive. All the isolated bacteria were tested for their sensitivity to selected antimicrobials.

In this study, we observed significant antibiotic resistance patterns among different bacterial strains. *Escherichia coli* exhibited high resistance to penicillin antibiotics, such as ampicillin (100%) and amoxicillin (96%), as well as cephalosporins, with cefixime at 100% and cefepime at 92%. Additionally, 96% of *E. coli* isolates displayed resistance to other antibiotics in the cephalosporin group. Notably, *E. coli* showed substantial sensitivity to colistin (92%) and tigecycline (72%). However, aminoglycosides and carbapenems demonstrated only moderate sensitivity.

While the sensitivity to colistin (88%) and tigecycline (72%), as well as sulfonamide (cotrimoxazole 34%), appeared promising, an alarming 99% of the *Acinetobacter* spp. isolates exhibited resistance to several antibiotic classes, including penicillin, cephalosporin, and carbapenem. This resistance pattern is particularly concerning for *Acinetobacter* spp.

*Klebsiella* spp., on the other hand, showed mixed effectiveness against various antibiotics, including colistin, tigecycline, gentamicin, and imipenem. These antibiotics displayed high to moderate sensitivity, but *Klebsiella* spp. exhibited significant resistance to penicillin, cephalosporin, and monobactam.

*Pseudomonas* spp. demonstrated potential sensitivity to colistin (84%) and tazobactam/piperacillin (61%) but were moderately sensitive to aminoglycosides, monobactams, carbapenems, and cefepimes. Conversely, *Pseudomonas* spp. showed high resistance against most antibiotics in the penicillin, cephalosporin, and fluoroquinolone groups.

*Staphylococcus aureus* exhibited susceptibility to various antibiotics, including vancomycin (100%), netilmicin (81%), and linezolid (81%). It was moderately sensitive to fusidic acid (75%), tigecycline (75%), cotrimoxazole (69%), gentamicin (63%), imipenem (56%), amikacin (56%), and meropenem (50%). However, it displayed high resistance to ampicillin (88%), cefixime (88%), cefotaxime (75%), ceftazidime (75%), and ceftriaxone (75%), and was moderately resistant to cefepime (69%), cefuroxime (69%), cephalexin (69%), levofloxacin (69%), and amoxiclav (63%).

Furthermore, the frequency of other bacterial strains, such as *Streptococcus* spp. (6), *Enterococcus* spp. (4), *Enterobacter* spp. (2), *Moraxella* spp. (2), *Proteus* spp. (2), and additionally *Staphylococcus* spp. (2) was low. Their sensitivity profile is given in Supplementary Table S1. Among the isolated microorganisms, *Acinetobacter* spp. and *Pseudomonas* spp. were notably multidrug resistant (99% and 92%, respectively), while *Acinetobacter* spp. and *Pseudomonas* spp. were seen to be extensively drug resistant (60% and 58%, respectively) (Table 4). In contrast, only 31% of *Staphylococcus aureus* strains exhibited multidrug resistance. These findings underscore the complex landscape of antibiotic resistance across various bacterial species, emphasizing the importance of responsible antibiotic use in clinical practice.

**Figure 2:**
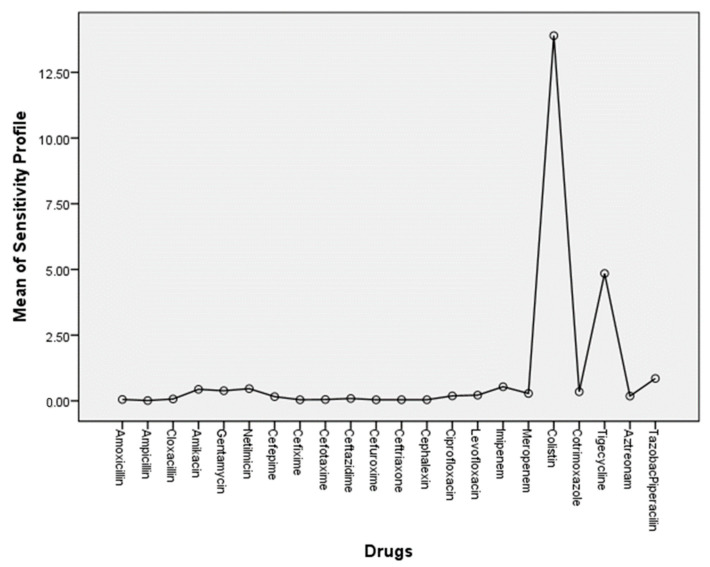
Most effective drugs against gram-negative bacteria according to Tukey’s post hoc comparison.

To identify the usefulness of the drugs used, we executed a one-way ANOVA test followed by Tukey’s post hoc test, using the sensitivity/resistant ratio within the groups and among the groups (*Acinetobacter* spp., *Klebsiella* spp., *E. coli*, and *Pseudomonas* spp. were considered as gram-negative group). We used the gram-negative panel drugs for the gram-negative isolates and gram-positive panel drugs for the gram-positive isolates for the calculation (Table S2). In both cases, we found that differences among the groups were statistically significant (*p* < 0.05). The plot (Figure 2) showed that colistin and tigecycline were the most effective drugs against gram-negative isolates in in vitro assays.

However, in the case of gram-positive organisms other than *Staphylococcus aureus* (*N* = 16), the number of isolates for other bacteria was relatively low, including *Streptococcus* spp. (*N* = 6), *Enterococcus* spp. (*N* = 4), and *Staphylococcus* spp. (*N* = 2). Due to the limited sample size, it wasn’t possible to carry out the ANOVA for the gram-positive organisms. However, after analyzing the sensitivity status of *Staphylococcus aureus*, we found that linezolid, vancomycin, fusidic acid, and tigecycline were the most effective drugs against the isolated gram-positive organisms in in vitro assays.

## 3. Discussion

This study found *Acinetobacter* spp., *Klebsiella* spp., *Pseudomonas* spp., *E. coli*, *Candida* spp., and *Staphylococcus aureus* to be the most prevalent microorganisms that spread nosocomial infections in one ICU setting of an urban hospital. In comparison to *Candida* spp., the presence of other fungi was minimal. These results provide crucial information for healthcare workers and public health officials to combat diseases caused by the microbes mentioned above. Additional findings from this study also show that more than 90% of the isolates were resistant to multiple classes of antibiotics. However, drugs like colistin (polymyxin) and tigecycline have demonstrated high sensitivity against isolated *Klebsiella* spp., *E. coli*, and *Acinetobacter* spp. Considering high antibiotic resistance, these findings are crucial in analyzing which antibiotics to prescribe to immunocompromised patients. The high presence of infectious microorganisms in the participant’s throats may be due to ventilation-mediated transfer, poor management, and compromised disinfection of ICU equipment, which is valuable data in helping the hospital management design better safety protocols to control the spread of such diseases.

Furthermore, the results of this study showed that *Acinetobacter* spp. was the most prevalent (34.07%) bacteria, and the isolates were mostly multidrug resistant (98.92%). Almost all the isolated *Acinetobacter* spp. were resistant to penicillin and the third generation of cephalosporin. Over 90% of the isolated *Acinetobacter* spp. were resistant to aminoglycoside, fluoroquinolones, carbapenem, monobactam, and tazobactam/piperacillin. Additionally, a high percentage of isolated *Acinetobacter* spp., despite their resistance pattern to other antibiotics, were susceptible to colistin—a drug of the polymyxin group and tigecycline (glycylcycline group). Moderate levels of sensitivity were also found against cotrimoxazole. The increasing trend of carbapenem resistance in *Acinetobacter* spp. is due to naturally producing β-lactamases, acquired β-lactamases like metallo-β-lactamases, carbapenem-hydrolyzing oxacillinases (CHDLs) enzyme, loss of outer membrane porin protein, and occasionally modification of penicillin-binding protein [8,21]. The adaptability demonstrated by *Acinetobacter* spp. has raised significant concerns, as it constrains the available therapeutic choices for patients in intensive care units (ICUs) [8]. This study showed similar findings in terms of microbe prevalence and antibiotic-resistant patterns as other studies. For instance, a retrospective study by Dereli et al. and studies from Bangladesh, Jamil et al., and Jesmin et al. [7,11,22] also reported a similar prevalence of microorganisms. Again, many studies from Bangladesh, India, Nepal, and other countries also reported similar findings regarding antibiotic resistance [8,11,23,24,25]. One prospective study from Morocco, for example, also reported 100% resistance of *Acinetobacter baumannii* to imipenem [26]; this resistance rate significantly exceeds our findings. The variation in these findings holds significant implications, as it highlights geographical disparities in antibiotic resistance. This suggests that resistance levels can vary based on the region, thereby presenting fresh challenges for healthcare professionals. Further epidemiological investigations are imperative to assess diverse resistance patterns across different geographic areas thoroughly.

Additional findings of this study, as mentioned above, include high percentages of gram-negative *Klebsiella* spp. and *E. coli* among Enterobacteriaceae. Consistent with these findings, earlier studies have also identified *Klebsiella pneumoniae*, a highly significant gram-negative bacterium within the *Klebsiella* spp., as the primary opportunistic pathogen affecting hospitalized individuals [27]. This is especially prominent among immunocompromised patients in intensive care units [28]. Other various international studies have also proven the prevalence of *Klebsiella* spp. and *E. coli* at ICUs globally [16,18,23,29,30]. Though the isolated *Klebsiella* spp. were susceptible to colistin and tigecycline, previous studies from Egypt and Brazil showed that imprudent use of these antibiotics can increase resistance against *Klebsiella* spp. [27,31,32,33]. For the other significant Enterobacteriaceae in this study, *E. coli*, more than 95% were multidrug resistant in nature, and almost all the organisms were resistant to tested penicillins and third-generation cephalosporin antibiotics. Previous research has also found high levels of resistant *E. coli* against carbapenem, cephalosporin, and tazobactam/piperacillin [7]. However, based on in vitro assays, our study revealed that colistin and tigecycline can be effective against *E. coli*. A high sensitivity against these two drugs shows the light to fight against nosocomial infections in ICU settings in urban hospitals. This finding is in agreement with a previous study [7], which also found colistin to be the most effective antibiotic against isolated multidrug-resistant *E. coli*.

Another important finding in relation to colistin is that it proved to be the only drug to which most of the isolated (84.21%) *Pseudomonas* spp. showed high sensitivity. Similarly, almost two thirds of the organism was sensitive to tazobactam/piperacillin. Previous studies by Jamil et al. and Jesmin et al. had similar findings [7,11] for tazobactam/piperacillin. However, in the case of sensitivity against colistin, this study’s findings were higher than Jesmin et al. [9] but similar to Jamil et al. [7]. This study observed a mixed resistance and sensitive status against aminoglycosides, imipenem, and aztreonam, with the results aligning with previous studies from Nepal, Egypt, and Iran [28,34,35], proving similar resistance patterns of the bacteria mentioned above despite geographical differences.

Among the gram-positive isolates, *Staphylococcus aureus* was the most prevalent organism at the ICU, among which almost one third were multidrug resistant (MDR), which is in conjunction with previous research [8]. This study found that all isolated *Staphylococcus aureus* were sensitive to vancomycin, netilmicin, linezolid, tigecycline, and fusidic acid. A study from Nepal also reported 100% sensitivity of *Staphylococcus aureus* to vancomycin [8], while a different academic paper from Egypt reported sensitivity against tigecycline (100%) in 2016, which is noticeably higher than this study (75%) [34]. These phenomena crucially show that antibiotic resistance varies with time, geographical location, socioeconomic status, study design, and study participants, proving the importance of repeated investigations into ever-changing antibiotic resistance patterns [5,36,37]. Additionally, the *Staphylococcus aureus* isolates showed mixed susceptibility to aminoglycosides, carbapenems, and tazobactam/piperacillin, where around half of the organisms were resistant and half were sensitive to those drugs. Resistance against aminoglycosides, amikacin (37.5%), and gentamicin (31.5%) were close to that found in a previous review article from Ethiopia [36], whereas sensitivity against gentamicin (62.5%) was found to be slightly higher than a previous study (54.55%) by Jesmin et al. [11].

Other than the aforementioned gram-positive and gram-negative bacteria, this study also found a significant number of isolated fungi, most of which were *Candida* spp. (7.33%). This high prevalence of *Candida* spp. may be due to the presence of underlying conditions of the admitted patient, like poor nutritional status, diabetes mellitus, and the use of steroids and broad-spectrum antibiotics—the findings of which align with previous studies [7]. *Candida* spp. is recognized as an opportunistic pathogen that often establishes colonization in patients. Distinguishing whether *Candida* spp. is merely colonizing or actively infecting patients is of utmost importance. *Candida* spp. has the potential to cause infections, particularly in individuals with compromised immune systems, a vulnerability frequently observed among patients in the intensive care unit (ICU) [7,14]. In light of these significant considerations, this study has opted to detect the presence of *Candida* spp. within the ICU environment. The high prevalence of multidrug-resistant bacteria is a global concern as our arsenal against these pathogens is gradually decreasing. Previous studies have shown that there are multiple ICU risk factors that have a significant role to play in the spread of nosocomial infections. Even though investigating these risk factors was beyond the scope of this study, it is still worth mentioning. A cohort study conducted in Turkish hospitals suggested that factors like the length of ICU stay and frequency and type of invasive procedures had a massive role to play in nosocomial infection spread [38]. The most isolated organism found from ICUs in the aforementioned study was *P. aeruginosa*, which provides dissimilar results to our study. The results of that study are similar to a study conducted in East India. Another study analyzed different ICU risk factors contributing to the spread of dangerous nosocomial infections and found that lung infections were the most common, with pneumonia being the most prevalent infection [39]. That study also found a correlation between the length of ICU stay and the probability of acquired infections. Although such investigative methods are beyond the scope of the conclusion our study achieves to reach, it is important to highlight potential ICU risk factors that lead to the prevalence of such infections.

This study highlights the methods through which these infections may be transmitted in hospitals, along with the differences in resistance patterns of microbes based on location, time, and study samples, compared with previous papers. In a world where nosocomial infections are becoming increasingly difficult to treat, affecting overall healthcare quality, especially for immunocompromised and elderly individuals, it is of utmost importance to equip healthcare facilities and the global scientific community with varied data in order to decide the best course of action in our everlasting battle against antimicrobial resistance.

### Limitations

A limitation of our study lies in our capacity to solely detect the presence of *Candida* spp. without the ability to definitively discern whether the isolated strains had the potential for colonization or were actively causing infections. Therefore, it is imperative for readers to bear this limitation in mind while interpreting our findings.

## 4. Materials and Methods

### 4.1. Study Design and Site

A cross-sectional study was conducted to analyze the presence of drug-resistant pathogens in the trachea of patients admitted to the ICU from 19 April 2021 to 21 March 2022, at the ICU facility of Ibn SINA Diagnostic and Imaging Center in Dhaka, Bangladesh.

### 4.2. Study Population and Sample Collection

A total of 200 tracheal aspiration samples were collected from endotracheal tube (ET tube) connectors of ICU patients by following the guidelines described by Irwin and Pratter [40].

This process involves the insertion of a fine plastic tube through a needle that is carefully placed into the trachea via the cricothyroid membrane. This catheter gently stimulates the cough reflex, facilitating the collection of respiratory secretions. A saline solution may be optionally introduced through the catheter to assist in the process.

### 4.3. Culture and Identification of Organisms

Collected specimens were handled aseptically, and those specimens were cultured into the designated media within two hours of collection. Specimens were streaked to three different agar media: blood agar, MacConkey agar, and chocolate agar. After 24 h of incubation at 37 °C, all the culture media were observed to determine whether there was growth. In the case of no growth, it was left for another 24 h for incubation under the same conditions. The second examination was carried out after 48 h of incubation. Suspected colonies selected based on colony morphology were then cultured on selective media. Colony morphology was observed, and biochemical tests were performed to identify the organisms. In the cases of polymicrobial growth on culture media, based on colony morphology, all the unique isolates were subjected to biochemical tests for identification. Bergey’s Manual of Systematics of Archaea and Bacteria was followed to identify the bacteria [41]. The different selective media that were used and the colony morphologies are presented in Table S3. The criteria for the interpretation of biochemical tests are presented in Supplementary Table S4.

After observing growth at the first phase in different media, fungi were detected by microscopic observation and inoculated in Sabouraud’s Dextrose Agar (SDA) for better growth, as proper growth of fungi cannot be observed within the short incubation period. These media were left for another 21 days to form a colony.

### 4.4. Antimicrobial Susceptibility Test

The antibiotic susceptibility test was performed following the Kirby–Bauer disc diffusion protocol, and the zones of inhibition were interpreted according to the CLSI guidelines. Briefly, the identified microorganisms were plated as a lawn in Mueller Hinton Agar (MHA) and left with antibiotic discs. After 24 h of incubation at 37 °C, the plate was observed to identify if the organisms were sensitive, intermediate, or resistant to the antibiotic. Table S2 lists the names of the antibiotics used for the antimicrobial susceptibility test against gram-negative and gram-positive bacteria.

### 4.5. Analysis

Multidrug resistance was defined according to the standardized international terminology to describe acquired resistance profiles in multidrug-resistant organisms (MDROs). A group of international experts created this terminology through a joint initiative between the ECDC (European Center for Disease Control) and the CDC (Center for Diseases Control). MDROs have been divided into three categories depending on their resistance profile: 1. MDROs—non-susceptible to at least one agent in three antimicrobial categories; 2. extensively drug-resistant (XDR) organisms non-susceptible to at least one agent in all but two or fewer antimicrobial categories; and 3. pan-drug-resistant (PDR) organisms—non-susceptible to all agents in all antimicrobial categories [1,13]. In this study, an organism was considered not MDR if it was not resistant to at least one agent of a minimum of three antimicrobial categories, and hence, named single drug resistant (SDR).

For our data analysis, we utilized IBM SPSS Statistics 20. We conducted a descriptive analysis to investigate age-related differences and the distribution of microorganisms. Additionally, to evaluate the effectiveness of individual drugs, we used one-way ANOVA and Tukey’s post hoc while taking into account variations within and between groups.

## Figures and Tables

**Table 1:** Age-wise distribution of most prevalent microorganisms.

Age Interval (Years)	Percentage Distribution among Age Groups	Number of Patients (*N* = 200) (%)	*Acinetobacter* spp. (*N* = 93) (%)	*Klebsiella* spp. (*N* = 60) (%)	*E. coli* (*N* = 25) (%)	*Pseudomonas* spp. (*N* = 38) (%)	*Staphylococcus aureus* (*N* = 16) (%)
13–19	1	2 (1)	2 (2.2)	1 (1.7)	0 (0)	1 (2.6)	0 (0)
20–29	3	6 (3)	2 (2.2)	2 (3.3)	0 (0)	1 (2.6)	0 (0)
30–39	9	19 (9.5)	12 (12.9)	2 (3.3)	2 (8.0)	2 (5.3)	1 (6.3)
40–49	7	14 (7)	4 (4.3)	3 (5.0)	1 (4.0)	3 (7.9)	3 (18.8)
50–59	16	32 (16)	16 (17.2)	10 (16.7)	4 (16)	7 (18.4)	3 (18.8)
60–69	27	54 (27)	28 (30.1)	15 (25)	9 (36)	9 (23.7)	5 (31.3)
70–79	19	39 (19.5)	19 (20.4)	12 (20)	4 (16)	5 (13.2)	3 (18.8)
80–89	12	23 (11.5)	7 (7.5)	9 (15)	4 (16)	7 (18.4)	1 (6.3)
90–99	6	11 (5.5)	3 (3.2)	6 (10)	1 (4.0)	3 (7.9)	0 (0)

**Table 2:** Isolated organisms.

Gram Stain Type	Isolated Organisms	Number of Isolated Organisms *N* (%)
Gram-negative	*E. coli*	25 (9.2)
	*Pseudomonas* spp.	38 (14)
	*Klebsiella* spp.	60 (22)
	*Moraxella* spp.	2 (0.7)
	*Enterobacter* spp.	2 (0.7)
	*Acinetobacter* spp.	93 (34)
	*Proteus* spp.	2 (0.7)
Gram-positive	*Staphylococcus aureus*	16 (5.9)
	*Staphylococcus* spp.	2 (0.7)
	*Streptococcus* spp.	6 (2.2)
	*Enterococcus* spp.	4 (1.5)
Fungi	*Candida* spp.	20 (7.3)
	Fungi	1 (0.4)
	Saprophytic fungi	2 (0.7)
	Total	273 (100)

**Table 3:** Sensitivity profile of the most prevalent isolated organisms.

Gram-Negative Microorganisms Number of (%) Isolates													Gram-Positive Microorganisms Number of (%) Isolates		
Antibiotics	*Acinetobacter* spp.			*Klebsiella* spp.			*E. coli*			*Pseudomonas* spp.			*Staphylococcus aureus*		
	(*N* = 93)			(*N* = 60)			(*N* = 25)			(*N* = 38)			(*N* = 16)		
	S	I	R	S	I	R	S	I	R	S	I	R	S	I	R
Penicillin															
Amoxicillin	1 (1.1)	0	92 (98.9)	7 (11.7)	0	53 (88.3)	1 (4)	0	24 (96)	1 (2.6)	0	37 (97.4)	7 (43.8)	0	9 (56.3)
Ampicillin	1 (1.1)	0	92 (98.9)	2 (3.3)	0	58 (96.7)	0	0	25 (100)	0	0	38 (100)	2 (12.5)	0	14 (87.5)
Cloxacillin	3 (3.2)	0	90 (96.8)	8 (13.3)	0	52 (86.7)	1 (4)	0	24 (96)	2 (5.3)	0	36 (94.7)	8 (50)	0	8 (50)
Amoxiclav	1 (1.1)	0	92 (98.9)	7 (11.7)	0	53 (88.3)	1 (4)	0	24 (96)	1 (2.6)	0	37 (97.4)	6 (37.5)	0	10 (62.5)
Aminoglycoside															
Amikacin	5 (5.4)	3 (3.2)	85 (91.4)	23 (38.3)	0	37 (61.7)	8 (32)	1 (4)	16 (64)	12 (31.6)	5 (13.2)	21 (55.2)	1 (6.2)	9 (56.3)	6 (37.5)
Gentamicin	7 (7.5)	0	86 (92.5)	24 (40)	0	36 (60)	5 (20)	0	20 (80)	13 (34.2)	1 (2.6)	24 (63.2)	10 (62.5)	1 (6.3)	5 (31.2)
Netilmicin	9 (9.7)	0	84 (90.3)	23 (38.3)	1 (1.7)	36 (60)	11 (44)	0	14 (56)	9 (23.7)	1 (2.6)	28 (73.8)	13 (81.2)	0	3 (18.8)
Cephalosporin															
Cefepime	2 (2.2)	1 (1.1)	90 (96.8)	7 (11.7)	0	53 (88.3)	2 (8)	0	23 (92)	10 (26.3)	3 (7.9)	25 (65.8)	5 (31.3)	0	11 (68.8)
Cefixime	0	1 (1.1)	92 (98.9)	6 (10)	0	54 (90)	0	0	25 (100)	2 (5.3)	0	36 (94.7)	2 (12.5)	0	14 (87.5)
Cefotaxime	0	1 (1.1)	92 (98.9)	7 (11.7)	1 (1.7)	52 (86.7)	1 (4)	0	24 (96)	1 (2.6)	0	37 (97.4)	2 (12.5)	2 (12.5)	12 (75)
Ceftazidime	1 (1.1)	0	92 (98.9)	8 (13.3)	0	52 (86.7)	0	1 (4)	24 (96)	6 (15.8)	1 (2.6)	31 (81.6)	2 (12.5)	2 (12.5)	12 (75)
Cefuroxime	1 (1.1)	0	92 (98.9)	6 (10)	0	54 (90)	1 (4)	0	24 (96)	0	0	38 (100)	5 (31.2)	0	11 (68.8)
Ceftriaxone	0	1 (1.1)	92 (98.9)	7 (11.7)	0	53 (88.3)	1 (4)	0	24 (96)	0	0	38 (100)	4 (25)	0	12 (75)
Cephalexin	1 (1.1)	0	92 (98.9)	5 (8.3)	1 (1.7)	54 (90)	1 (4)	0	24 (96)	1 (2.6)	0	37 (97.4)	4 (25)	1 (6.2)	11 (68.8)
Fluoroquinolones															
Ciprofloxacin	4 (4.3)	0	89 (95.7)	16 (26.7)	2 (3.3)	42 (70)	3 (12)	0	22 (88)	6 (15.8)	1 (2.63)	31 (81.6)	6 (37.5)	1 (6.3)	9 (56.3)
Levofloxacin	5 (5.4)	1 (1.1)	87 (93.6)	18 (30)	2 (3.3)	40 (66.7)	3 (12)	0	22 (88)	7 (18.4)	0	31 (81.6)	5 (31.2)	0	11 (68.8)
Carbapenem															
Imipenem	7 (7.5)	1 (1.1)	85 (91.4)	24 (40)	6 (10)	30 (50)	11 (44)	0	14 (56)	12 (31.6)	1 (2.6)	25 (65.8)	9 (56.2)	0	7 (43.8)
Meropenem	2 (2.2)	0	91 (97.9)	20 (33.3)	1 (1.7)	39 (65)	6 (24)	0	19 (76)	8 (21.1)	1 (2.6)	29 (76.3)	8 (50)	0	8 (50)
Polymyxin															
Colistin	82 (88.2)	3 (3.2)	8 (8.6)	57 (95)	1 (1.7)	2 (3.3)	23 (92)	0	2 (8)	32 (84.2)	0	6 (15.8)			
Sulfonamide															
Cotrimoxazole	32 (34.4)	8 (8.6)	53 (57)	18 (30)	1 (1.7)	41 (68.3)	4 (16)	0	21 (84)	5 (13.2)	0	33 (86.8)	11 (68.7)	0	5 (31.3)
Oxazolidinone															
Linezolid													13 (81.3)	0	3 (18.7)
Glycopeptide															
Vancomycin													16 (100)	0	0
Glycylcycline															
Tigecycline	67 (72.0)	15 (16.1)	11 (11.8)	42 (70)	12 (20)	6 (10)	18 (72)	4 (16)	3 (12)	8 (21.1)	3 (7.9)	27 (71)	12 (75)	0	4 (25)
Fusidane															
Fusidic Acid													12 (75)	0	4 (25)
Monobactam															
Aztreonam	1 (1.1)	2 (2.2)	90 (96.8)	7 (11.7)	1 (1.7)	52 (86.7)	1 (4)	0	24 (96)	13 (34.2)	1 (2.6)	24 (63.2)			
Beta-lactamase inhibitor + penicillin															
Tazobactam + Piperacillin	2 (2.2)	2 (2.2)	89 (95.6)	19 (31.7)	1 (1.7)	40 (66.7)	6 (24)	2 (8)	17 (68)	23 (60.5)	6 (15.8)	9 (23.7)	7 (43.7)	0	9 (56.3)

S: sensitive; I: intermediate; R: resistance.

**Table 4:** Resistance status of the prevalent organisms *.

Isolated Organism	SDR	MDR
	*N* (%)	*N* (%)
*Acinetobacter* spp.	1 (1.08)	92 (98.92)
*Pseudomonas* spp.	3 (7.89)	35 (92.09)
*Klebsiella* spp.	6 (9.99)	54 (90.01)
*Staphylococcus aureus*	11 (68.75)	5(31.25)
*E. coli*	1 (4.00)	24 (96.00)

* SDR is Single Drug-Resistant and MDR is Multidrug-resistant. We did not test all the antibiotics required to declare XDR (Extensively drug-resistant) and PDR (Pan drug-resistant).

## Data Availability

The data presented in this study are available on request from the corresponding author. The data are not publicly available due to ethical restrictions.

## Supplementary Materials

The following supporting information can be downloaded at: https://www.mdpi.com/article/10.3390/antibiotics13010062/s1, Table S1: Sensitivity profile of the less prevalent isolated organisms; Table S2: Antimicrobial panels; Table S3: Colony morphology of specific bacteria on selective media; Table S4: Biochemical test interpretation for different organisms. References [42,43,44,45,46,47,48,49,50,51,52,53,54,55,56,57,58,59,60,61] are cited in supplementary materials.
