# Supplementary material for: High Prevalence of Multidrug-Resistant Bacteria in the Trachea of Intensive Care Units Admitted Patients: Evidence from a Bangladeshi Hospital
Source: Antibiotics (Basel). 2024 Jan 8;13(1):62. doi: 10.3390/antibiotics13010062 (PMC10812536; doi:10.3390/antibiotics13010062)
Supplement: Supplementary file 1 [file antibiotics-13-00062-s001.zip › antibiotics-2619287-supplementary.pdf]

Supplementary tables:

Table S1. Sensitivity profile of the less prevalent isolated organisms: The degree of sensitivity exhibited by the bacterial isolates to the antibiotics is shown. The number enclosed by the first bracket is the percentage of isolates.

**Antibiotics**	***Streptococcus* spp.** *N* = 6			***Enterococcus* spp.** *N* = 4			***Enterobacter* spp.** *N* = 2			***Moraxella* spp.** *N* = 2			***Proteus* spp.** *N* = 2			***Staphylococcus* spp.** *N* = 2		
	S	I	R	S	I	R	S	I	R	S	I	R	S	I	R	S	I	R
**Penicillin**																		
Amoxicillin	4 (66.7)	0	2 (33.3)	3 (75)	0	1 (25)	1 (50)	0	1 (50)	1 (50)	0	1 (50)	1 (50)	0	1 (50)	0	0	2 (100)
Ampicillin	4 (66.7)	0	2 (33.3)	3 (75)	0	1 (25)	1 (50)	0	1 (50)	1 (50)	0	1 (50)	1 (50)	0	1 (50)	0	0	2 (100)
Cloxacillin	3 (50)	0	3 (50)	3 (75)	0	1 (25)	1 (50)	0	1 (50)	0	0	2 (100)	1 (50)	0	1 (50)	1 (50)	0	1 (50)
Amoxiclav	5 (83.3)	0	1 (16.7)	3 (75)	0	1 (25)	2 (100)	0	0	2 (100)	0	0	1 (50)	0	1 (50)	0	0	2 (100)
**Aminoglycoside**																		
Amikacin	1 (16.7)	0	5 (83.3)	1 (25)	1 (25)	2 (50)	0	1 (50)	1 (50)	1 (50)	0	1 (50)	2 (100)	0	0	1 (50)	0	1 (50)
Gentamicin	1 (16.7)	2 (33.3)	3 (50)	1 (25)	1 (25)	2 (50)	2 (100)	0	0	2 (100)	0	0	1 (50)	0	1 (50)	1 (50)	0	1 (50)
Netilmicin	3 (50)	1 (16.7)	2 (33.3)	0	1 (25)	3 (75)	2 (100)	0	0	1 (50)	0	1 (50)	1 (50)	0	1 (50)	1 (50)	0	1 (50)
**Cephalosporin**																		
Cefepime	4 (66.7)	0	2 (33.3)	3 (75)	0	1 (25)	1 (50)	0	1 (50)	1 (50)	0	1 (50)	2 (100)	0	0	1 (50)	0	1 (50)
Cefixime	2 (33.3)	2 (33.3)	2 (33.3)	1 (25)	0	3 (75)	0	0	2 (100)	1 (50)	0	1 (50)	1 (50)	0	1 (50)	0	0	2 (100)
Cefotaxime	3 (50)	0	3 (50)	3 (75)	0	1 (25)	1 (50)	0	1 (50)	1 (50)	0	1 (50)	1 (50)	0	1 (50)	0	0	2 (100)
Ceftazidime	3 (50)	0	3 (50)	3 (75)	0	1 (25)	0	0	2 (100)	1 (50)	0	1 (50)	2 (100)	0	0	0	0	2 (100)
Cefuroxime	3 (50)	0	3 (50)	2 (50)	1 (25)	1 (25)	1 (50)	0	1 (50)	2 (100)	0	0	1 (50)	0	1 (50)	1 (50)	0	1 (50)
Ceftriaxone	3 (50)	0	3 (50)	3 (75)	0	1 (25)	1 (50)	0	1 (50)	1 (50)	0	1 (50)	1 (50)	0	1 (50)	1 (50)	0	1 (50)
Cephalexin	0	1 (16.7)	5 (83.3)	3 (75)	0	1 (25)	1 (50)	0	1 (50)	1 (50)	0	1 (50)	1 (50)	0	1 (50)	0	0	2 (100)
**Fluoroquinolones**																		

Antibiotics	*Streptococcus* spp. *N* = 6			*Enterococcus* spp. *N* = 4			*Enterobacter* spp. *N* = 2			*Moraxella* spp. *N* = 2			*Proteus* spp. *N* = 2			*Staphylococcus* spp. *N* = 2		
	S	I	R	S	I	R	S	I	R	S	I	R	S	I	R	S	I	R
Ciprofloxacin	2 (33.3)	2 (33.3)	2 (33.3)	2 (50)	0	2 (50)	1 (50)	1 (50)	0	1 (50)	0	1 (50)	1 (50)	0	1 (50)	1 (50)	0	1 (50)
Levofloxacin	3 (50)	0	3 (50)	3 (75)	0	1 (25)	1 (50)	0	1 (50)	2 (100)	0	0	1 (50)	0	1 (50)	0	0	2 (100)
**Carbapenem**																		
Imipenem	4 (66.7)	0	2 (33.3)	3 (75)	0	1 (25)	2 (100)	0	0	1 (50)	0	1 (50)	2 (100)	0	0	1 (50)	0	1 (50)
Meropenem	4 (66.7)	0	2 (33.3)	3 (75)	0	1 (25)	2 (100)	0	0	1 (50)	0	1 (50)	1 (50)	0	1 (50)	0	0	2 (100)
**Polymyxin**																		
Colistin	4 (66.7)	0	2 (33.3)	3 (75)	0	1 (25)	1 (50)	0	1 (50)	1 (50)	0	1 (50)	2 (100)	0	0			
**Sulfonamide**																		
Cotrimoxazole	1 (16.7)	1 (16.7)	4 (66.7)	0	0	4 (100)	1 (50)	0	1 (50)	2 (100)	0	0	1 (50)	0	1 (50)	1 (50)	0	1 (50)
**Oxazolidinone**																		
Linezolid																1 (50)	0	1 (50)
**Glycopeptide**																		
Vancomycin																1 (50)	0	1 (50)
**Tigecycline**																		
Tigecycline	6 (100)	0	0	4 (100)	0	0	2 (100)	0	0	1 (50)	0	1 (50)	2 (100)	0	0	0	1 (50)	1 (50)
**Fusidane**																		
Fusidic Acid																1 (50)	0	1 (50)
**Monobactam**																		
Aztreonam	3 (50)	0	3 (50)	3 (75)	0	1 (25)	1 (50)	0	1 (50)	1 (50)	0	1 (50)	2 (100)	0	0			
**Beta-lactamase inhibitor + pencillin**																		
Tazobactam + Piperacillin	6 (100)	0	0	3 (75)	0	1 (25)	1 (50)	0	1 (50)	1 (50)	0	1 (50)	2 (100)	0	0	0	0	2 (100)

S: sensitive; I: intermediate; R: resistance

Table S2. Antimicrobial panels: The antibiotic class, the name of the antibiotics, and their spectrum is mentioned.

Drug groups	Drugs	Gram-negative panel	Gram-positive panel
Penicillin	Amoxicillin	✓	✓
	Ampicillin	✓	✓
	Cloxacillin	✓	✓
	Amoxiclav		✓
Aminoglycoside	Amikacin	✓	✓
	Gentamycin	✓	✓
	Netilmicin	✓	✓
Cephalosporin	Cefepime	✓	✓
	Cefixime	✓	✓
	Cefotaxime	✓	✓
	Ceftazidime	✓	✓
	Cefuroxime	✓	✓
	Ceftriaxone	✓	✓
	Cephalexin	✓	✓
Fluoroquinolones	Ciprofloxacin	✓	✓
	Levofloxacin	✓	
Carbapenem	Imipenem	✓	✓
	Meropenem	✓	✓
Polymyxin	Colistin	✓	
Sulfonamide	Cotrimoxazole	✓	✓
Oxazolidinone	Linezolid		✓
Glycopeptide	Vancomycin		✓
Tigecycline	Tigecycline	✓	✓
Fusidane	Fusidic Acid		✓
Monobactam	Aztreonam	✓	
Beta-lactamase inhibitor + penicillin	Tazobactam + Piperacillin	✓	

Table S3. Colony morphology of specific bacteria on selective media.

Organism	Gram +ve/-ve	Media	Expected Colony Morphology	Reference
Escherichia coli	-ve	Eosin Methylene Blue agar (EMB)	Blue-black colonies with green metallic sheen	Basavaraju & Gunashree, 2023 [42]
Pseudomonas spp.	-ve	Cetrimide Agar	Yellow-green to blue-green colonies which exhibit fluorescence at 250 nm UV light	*Cetrimide Agar | Principle | Preparation | Results*, n.d. [43]
Klebsiella spp.	-ve	Klebsiella Blue Agar	Dark green	Prasad et al., 2022 [44]
Moraxella spp.	-ve	Blood agar	Smooth, flat, uniform, buff colonies	Public Health England, 2015 [45]
Enterobacter spp.	-ve	Eosin Methylene Blue agar (EMB)	Pink/buff color with darker centers	Libretexts, 2021 [46]
Acinetobacter spp.	-ve	CHROMagar Acinetobacter	Red colonies	Ajao et al., 2011 [47]
Proteus spp.	-ve	MacConkey agar	Smooth, pale, or colorless colonies	Tankeshwar & Tankeshwar, 2022 [48]
Staphylococcus aureus	+ve	Mannitol salt agar (MSA)	Yellow, round colonies	Missiakas & Schneewind, 2013 [49]
Staphylococcus spp.	+ve	Mannitol salt agar (MSA)	Yellow, round colonies	Missiakas & Schneewind, 2013 [49
Streptococcus spp.	+ve	Blood agar	Grayish-white, smooth, glossy, and translucent colonies	Hossain, 2014 [50]
Enterococcus spp.	+ve	M-enterococcus (ME) agar	Round, pink to dark maroon-colored colonies	"M-enterococcus (ME) Agar," 2003 [51]

Table S4. Biochemical test interpretation for different organisms.

Organism	Motility	Indole	Urease	Catalase	Oxidase	MR	VP	Citrate utilization	Glucose Ferm.	Sucrose Ferm.	Lactose Ferm.	Gas Prod.	H_2S Prod.	Hemolysis	Reference
Escherichia coli	+ve	+ve	-ve	+ve	-ve	+ve	-ve	-ve	+ve	variable	+ve	+ve	-ve	Alpha/ Beta/ Gamma	Aryal, 2022 [52]
Pseudomonas spp.	+ve	-ve	-ve	+ve	+ve	-ve	-ve	+ve	-ve	-ve	-ve	+ve	-ve	Beta	Aryal, 2022 [53]
Klesbiella spp.	-ve	-ve	+ve	+ve	-ve	-ve	+ve	+ve	+ve	+ve	+ve	+ve	-ve	Gamma	Aryal, 2022 [54]
Moraxella spp.	-ve	-ve	-ve	+ve	+ve	-ve		-ve	-ve	-ve	-ve	-ve	-ve	Gamma	Sheikh et al., 2020) [55]
Enterobacter spp.	+ve	-ve	-ve	+ve	-ve	-ve	+ve	+ve	+ve	+ve	+ve	+ve	-ve		Aryal, 2022 [56]
Acinetobacter spp.	-ve	-ve	-ve	+ve	-ve	-ve	-ve	+ve	+ve	-ve	variable	-ve	-ve	Gamma	Aryal, 2022 [57]
Proteus spp.	+ve	variable	+ve	+ve	-ve	+ve	-ve	variable	+ve	-ve	-ve	variable	+ve	Gamma/ Beta	Aryal, 2022 [58]
Staphylococcus aureus[*]	-ve	-ve	+ve	+ve	-ve	+ve	+ve	+ve	+ve	+ve	+ve	-ve	-ve	Beta	Aryal, 2022 [59]
Streptococcus spp.	-ve	-ve	-ve	-ve	-ve	+ve	-ve	+ve	+ve	+ve	+ve	-ve	-ve	Beta	Batra, 2020 [60]
Enterococcus spp.	-ve	-ve	-ve	-ve	-ve		+ve	-ve	+ve	variable	+ve	-ve	-ve	Gamma/ Alpha	Aryal, 2022 [61]

**Staphylococcus aureus* was further differentiated from the other *Staphylococcus spp.* by coagulase test. *Staphylococcus aureus* is coagulase positive. MR: Methyl red test; VP: Voges-Proskauer test
